# Modulation of the p75 neurotrophin receptor suppresses age-related basal forebrain cholinergic neuron degeneration

**DOI:** 10.1038/s41598-019-41654-8

**Published:** 2019-03-27

**Authors:** Youmei Xie, Rick B. Meeker, Stephen M. Massa, Frank M. Longo

**Affiliations:** 1 Department of Neurology, University of North Carolina, Chapel Hill, NC 27599 USA; 2 Department of Neurology, San Francisco Veterans Administration Health Care System and University of California San Francisco, San Francisco, CA 94121 USA; 3 Department of Neurology and Neurological Sciences, Stanford University School of Medicine, Stanford, CA 94305 USA

## Abstract

Age-related degeneration of basal forebrain cholinergic neurons (BFCNs) is linked to cognitive impairment. The p75 neurotrophin receptor (p75^NTR^) has been proposed to mediate neuronal degeneration in aging. Therefore, we tested the hypothesis that modifying p75^NTR^ function would prevent or reverse aging-related neuronal degeneration using LM11A-31, a small molecule p75^NTR^ modulator that downregulates degenerative and upregulates trophic receptor-associated signaling. Morphological analysis in mice showed loss of BFCN area detectable by 18 months of age. Oral administration of LM11A-31 from age 15 to 18 months resulted in a dose-related preservation of BFCN area and one month of treatment from 17 to 18 months also preserved cell area. To evaluate reversal of established neuronal atrophy, animals were treated from 21 to 25 months of age. Treatment was associated with an increase of cell size to a mean area larger than that observed at 18 months, accompanied by increases in mean MS/VDB neurite length, as well as increased cholinergic fiber density and synaptophysin pre-synaptic marker levels in the hippocampus. These findings support the idea that modulation of p75^NTR^ activity can prevent and potentially reverse age-associated BFCN degeneration. Moreover, this may be achieved therapeutically with orally bioavailable agents such as LM11A-31.

## Introduction

Basal forebrain cholinergic neurons (BFCNs) provide cholinergic input to broad regions of the cortex and hippocampus in rodents, humans and other mammals^1,2^ which promotes synaptic plasticity and supports mechanisms serving attention, working memory and processing speed^3–6^. The importance of this cholinergic system has engendered substantial interest in the implications for cognitive function of BFCN degeneration that occurs during normal aging^7–14^ and in prodromal or early stages of Alzheimer’s and other neurodegenerative diseases^10,11,14–22^, Indeed, BFCNs are particularly vulnerable to age-related degeneration^13^ and that process is one of the major mechanisms proposed to underlie age-associated memory impairment (AAMI)^5^.

Age-related degeneration of BFCNs is reflected in several morphological changes, including somal atrophy, decreases in neurite length and target innervation^9,23–25^ which have been found in aged rodents, non-human primates and humans. In some studies, age-related decreases in cholinergic innervation of hippocampal regions were not detected^26,27^. Measurements of BFCN numbers have been more variable, showing either no effect or decreases with aging^9,10,26,28,29^. In longitudinal volumetric MRI studies of healthy aging among cognitively intact human subjects, volume loss of basal forebrain structures is disproportionately greater than that occurring in overall grey matter^11^. Several molecular and functional changes have also been identified, including diminished neurofilament expression^23^, reduction of calbindin-D28K, Ca2^++^/calmodulin-dependent protein kinase I and growth-associated protein-43, increased activation of µ-calpain and L-type calcium channels^30^, and increased phosphorylation of tau^31^. Further, there are deficits in the induction and maintenance of LTP^32^.

Multiple observations suggest that changes in neurotrophin (NT) signaling have a substantial role in age-related BFCN degeneration. NTs consist of a family of protein ligands that include nerve growth factor (NGF), brain-derived neurotrophic factor (BDNF) and neurotrophin-3 (NT-3) that are also present in pro-forms^33,34^. Each of the mature forms bind to its cognate tyrosine kinase receptor (TrkA, TrkB and TrkC, respectively) and to the common neurotrophin receptor known as p75^NTR^, while the pro-forms also bind the dual receptor complex of p75^NTR^ and sortilin. Within the BFCN system, expression of TrkA and p75^NTR^ is relatively high compared with other CNS cell populations^29,35^. With concomitant binding to the Trk/p75^NTR^ receptors, or to Trk receptors alone, NTs in their mature forms generally have a neurotrophic effect. In its pro-form, NGF binding to p75^NTR^ with reduced or absent TrkA interaction, promotes degenerative signaling and neuronal degeneration/death^36,37^. The finding of increased levels of proNGF in the hippocampus in aged rats^38^ raises the possibility that proNGF/p75^NTR^ signaling contributes to age-related atrophy of neurons expressing p75^NTR^ including BFCNs innervating the hippocampus^39^. In several contexts, p75^NTR^ in its unliganded state also supports degenerative signaling suggesting a baseline anti-trophic effect. This is supported by studies in which transgenic mice lacking intact wild-type p75^NTR^, demonstrate increased size of BFCNs along with enhanced cholinergic innervation of the hippocampus, cortex and amygdala^29,40–44^. The ability of antisense-mediated knockdown of p75^NTR^ to enhance hippocampal cholinergic innervation further supports the findings from knockout studies^45^. Delivery of mature NGF to the CNS of aged rats^46–55^ and non-human primates^56–61^ results in reversal of age-related BFCN atrophy. However, in these studies of mature NGF, which functions through binding to both the TrkA and p75 receptors, the contributions of each receptor and their potential interactions were not evaluated. Thus, the potential for pharmacologically targeting p75^NTR^ alone as a therapy for aging-related cognitive decline remains unexplored.

p75^NTR^ small molecule ligands have been developed in attempts to both circumvent the pharmacokinetic shortcomings of large peptide ligands and to specifically target p75^NTR^ receptor mechanisms independently of other NT receptors^62–66^. One of these, LM11A-31, has been extensively characterized, and has been found to function as a p75^NTR^ receptor modulator (rather than a simple agonist or antagonist) to shift p75^NTR^ from neurodegenerative to neurotrophic signaling in neurons expressing p75^NTR^ while having no detectable effect on TrkA^64,67,68^. In addition, LM11A-31 has also been shown to block proNGF binding to and activation of p75^NTR^ receptor degenerative signaling^64,69^. In other studies, p75^NTR^-linked neurodegenerative signaling has been found to have a substantial overlap with neurodegenerative signaling likely to occur in Alzheimer’s disease (AD). LM11A-31 has been found to inhibit degenerative signaling in multiple *in vitro* and *in vivo* AD models^67,70–72^ and is currently undergoing testing in a human phase 2a trial in AD (NCT03069014). A key question regarding the beneficial effects of LM11A-31 in AD models is whether these effects include counteracting the contributions of age-related degeneration *per se*. Addressing this question requires its application in a non-AD, age-based degeneration model. Moreover, the finding of a clinically viable compound with this profile would introduce a candidate for the treatment of age-related BFCN degeneration and thus possibly age-related cognitive impairment. In the current study, we applied LM11A-31 to test the hypothesis that small molecule modulation of p75^NTR^ might slow or reverse BFCN neurodegeneration associated with natural aging.

## Materials and Methods

### Animals

Young adult and aged C57BL6 mice were purchased from Jackson labs and the National Institute of Aging colony (NIH, USA). These mice were housed individually at the University of North Carolina within a Division of Lab Animal Medicine-operated facility in a controlled, pathogen-free environment (constant temperature 22 ± 1 °C; humidity 50–60%; lights on 06:00–18:00 hr). Mice were housed with free access to food and water until the appropriate age (4, 9 and 11 months for young adult mice or 15, 18 and 24–25 months for aged mice). All experimental procedures were approved by the IACUC of the University of North Carolina and were conducted in accordance with the NIH Guide for the Care and Use of Laboratory Animals and compliance to PHS Policy on Humane Care and Use of Laboratory Animals.

### LM11A-31 administration

LM11A-31 was synthesized and purified in its HCl form to our specifications as previously described^67^. The compound was dissolved in deionized H_2_O to a concentration of 5 mg/ml and filter sterilized. Mice were randomly assigned to treatment or vehicle groups, were fasted overnight and LM11A-31 or an equivalent volume of vehicle (deionized water) was delivered once daily in the morning via oral gavage at a dose of 50 mg/kg (10 µl/g). In independent experiments with this dosing paradigm LM11A-31 reached a peak concentration of approximately 262 ng/ml or approximately 1.8 µM in brain with a t_1/2_ in brain of 3–4 hrs. At the 1 hr and subsequent time points the brain/plasma ratios were >1^70^.

LM11A-31 treatment was initiated at 11, 15, 17 or 21 months. Three groups of mice were included at each time point–a young (4 months) comparator cohort, and aged groups given LM11A-31 or vehicle alone, with treatment durations of 1–4 months. One day after the final dose, mice were deeply anesthetized and perfused with saline followed by a solution of 4% paraformaldehyde in 0.01 M PBS in preparation for histochemical analysis. Estimates of the median effective dose (EC_50_) were obtained from non-linear regression analysis using the Graphpad Prism 5 log agonist vs response model.

### Immunocytochemistry

Mice were euthanized under deep avertin anesthesia and perfused transcardially with phosphate buffered saline (PBS; 0.01 M phosphate buffer containing 0.9% sodium chloride, pH 7.4) followed by 4% paraformaldehyde in PBS). The brains were removed and post-fixed overnight (about 16 hr) in the same fixative. The brains were then soaked in 30% sucrose in PBS until they sank. Frozen coronal sections through the forebrain were cut on a sledge microtome (MICROM HM 430, Germany) at a thickness of 40 µM. Sections were stored in cryoprotectant at −20 °C until processing for immunocytochemistry.

Animals from each group were processed together for immunocytochemistry in parallel to provide matched controls and to minimize variation in staining. Every 6^th^ section through the region of the medial septum and diagonal band nuclei was immunostained for choline acetyltransferase.

Briefly, free floating sections were washed in PBS and incubated for 30 min at room temperature (RT) in PBS containing 3% hydrogen peroxide (H_2_O_2_) to quench endogenous peroxidase activity. Sections were washed in PBS, permeabilized with 0.4% Triton-X100 in PBS for 30 min at RT, and then blocked for 60 min with PBS containing 3**%** normal rabbit serum, 3% BSA and 0.1% Triton X-100 for 1 hr at 4 °C. Sections were transferred to polyclonal goat anti-ChAT (Chemicon AB144P) at a 1:1000 dilution in PBS + 0.1% Triton + 3% normal serum for 24 hr at 4 °C then were washed three times and incubated in a biotinylated rabbit anti-goat IgG (Chemicon AP106) at 1:500 for 2 hr at 4 °C. Sections were washed and developed in avidin-biotinylated peroxidase solution (Vector Laboratories ABC kit 6100) with intensified diaminobenzidine (DAB) as the substrate (Vector Laboratories SK4100). After a final wash in PBS and deionized water, the sections were mounted on Superfrost/Plus Microscope Slides (Fisher Scientific PA 15219), dried overnight at RT, dehydrated in absolute ethanol, cleared in xylene, and coverslipped with Mounting Medium #4111 (Richard-Allan Scientific, Kalamazoo MI). Slides from individual animals were randomized and coded so all subsequent analyses could be carried out in a blinded fashion with regard to age and treatment group.

### Immunofluorescence

Every sixth section from control and treated animals was washed in PBS, incubated in 0.4% Triton X-100 in PBS for 30 min at RT and then blocked for 60 min with 3**%** normal serum (Chemicon), 3% BSA (Sigma), and 0.1% Triton (Sigma) at 4 °C. Sections were transferred to polyclonal goat anti-ChAT (Chemicon/Millipore, AB144P) at 1:1000, or rabbit anti-MAP-2 (Millipore, AB5622) at 1:500 diluted in PBS + 0.1% Triton X100 + 3% normal serum for 24 hr at 4 °C. Visualization was accomplished using rabbit anti-goat Alexa488, donkey anti-mouse Alexa594 or donkey anti-rabbit Alexa594 (Molecular Probes Eugene, Oregon) at a dilution of 1:1000 for 1 hour at RT. Sections were rinsed in PBS and mounted on Superfrost/Plus Microscope Slides (Fisher Scientific PA 15219) with Fluoromount-G (SouthernBiotech 0100-01). Slides from individual animals were randomized and coded as above.

### Western blot analysis

Animals were deeply anaesthetized with avertin and perfused transcardially with cold 0.9% NaCl for 6 min. Cortex and hippocampus were dissected from the brain and homogenized in RIPA Lysis Buffer (Millipore, 20–188) containing 1% protease inhibitor cocktail (Sigma, P8340). Protein levels were determined by the Bradford protein assay (Biorad). Immunoblots were performed according to standard procedures. Briefly, 20 µg protein/lane were separated by sodium dodecyl sulfate-polyacrylamide gel electrophoresis (SDS-PAGE) and transferred to PVDF membranes (Invitrogen). Membranes were blocked for 1 h at room temperature with Tris buffered saline containing 0.2% Tween-20 and 5% milk powder, followed by an overnight incubation with mouse anti-synaptophysin (Millipore, MAB5258 1:1000) diluted in the blocking solution. After several rinses with Tris buffered saline/0.2% Tween 20, membranes were incubated with goat anti-mouse (1:10,000) conjugated to horseradish peroxidase (HRP) for 1 h at room temperature. To quantify the relative amount of proteins, the blots were stripped and re-probed with mouse anti-GAPDH, 1:2000 (Millipore, MAB374) for 1 h, followed by goat anti-mouse-HRP (1:10,000). Bands were visualized using SuperSignal West Pico Chemiluminescent Substrate (Thermo, 34080) and quantified with UN-SCAN-IT gel software (Silk Scientific Inc).

### Stereology

Digital images of the medial septum and diagonal band nuclei were collected on a Nikon MICROPHOT-FXA microscope equipped with a computer-controlled stepper motor stage that allowed movement in the x-, y- and z-axes. Images were collected and analyzed using Stereo Investigator 9.0 (MBF Bioscience Williston, VT. USA).

#### ChAT neuron size in the MS and VDB

The size of cholinergic neurons in MS and VDB regions was estimated from sections stained for choline acetyltransferase using diaminobenzidine (DAB) as the chromogen. All sections used for the stereological assessment were stained and processed together to minimize variability. Stereo Investigator 9.0 optical fractionator and vertical nucleator probes were used for the analysis. The individual performing the analysis was blinded to the experimental conditions. Anatomical boundaries of the *MS/VDB* were defined rostrally by the meeting of the corpus callosum at the midline, (1.10 mm anterior to bregma) and caudally by the midline crossing of the anterior commissure (0.14 mm anterior to bregma)^73^. MS and the VDB were traced in each section at low magnification using a 4x objective. Cell size measurements were collected using the following parameters: counting frame, 80 × 80 µm; guard zone, 5 µm; and counting depth, 20 µm. ChAT positive cell size was measured with a 100x objective, using criteria that fulfilled the unbiased counting rules. The vertical nucleator probe was used to estimate the area of the largest cross-sectional profile of each cholinergic neuron that fulfilled the 3D counting rules of the optical fractionator probe. At the largest cross-sectional profile of the cell, a set of four rays were generated forming two lines that crossed at a point within the cell and radiated with random orientations towards the edge of the profile. The four intersections with the cell boundary were marked. The area of the profiles (A) was estimated according to the equation $a=\pi {l}^{2}$ where *l* is the average length of the intercepts.

#### Cholinergic neuron population estimate

The total number of cholinergic neurons (N) was estimated in the same DAB stained sections as above with the *optical fractionator probe* (Stereo Investigator, MBF Bioscience)^74^ based on the formula: *N* = ∑*Q*^−^*∙* $\frac{t}{h}$ *∙* $\frac{1}{as{\int }^{}}\,$*∙* $\frac{1}{ss{\int }^{}}$ where *N* = total number of neurons, ∑*Q*^−^ = number of neurons actually counted in the specimens, *t* = section thickness calculated by the software at each sampling site, h = counting frame height (h = 30), ss∫ = section sampling fraction (ss**∫** = 1/6), and as**∫** = area sampling fraction (counting frame/sampling grid = 35um × 35um/80um × 80um). Although not the primary objective of the studies, the counts were used to indicate if cells were lost from the sample population under the various experimental conditions. The use of the optical fractionator probe provided an unbiased estimate of cell numbers independent of sample shrinkage. The area of the counting frame was 1225 µm^2^ in a total sampling grid area of 6400 µm^2^. The average thickness of the counting area was 30 µm. On average, 194.5 sampling sites were counted from 4 sections with 46.6 total markers for each region and a Gundersen coefficient of error ranging from 0.098 to 0.233 (mean = 0.1568).

#### Neurological analysis of ChAT neurite length in the MS and fiber density in the hippocampus and parietal cortex

Sections were visualized and tiled, Z-stack fluorescent images were captured with an LSM710 confocal microscope (Carl Zeiss Inc) from coronal sections through the MS; dorsal hippocampus including alveus, stratum oriens, stratum pyramidale (CA1), stratum radiatum and stratum lacunosum/moleculare; and M1 region parietal cortex, spanning layers 1 through 6. MBF Autoneuron was used to trace neurites attached to cholinergic somata and their length determined. MBF Autoneuron was also used to trace fiber length per tissue volume for hippocampal and cortical regions. Volumes analyzed for ChAT fiber density were defined using consistent hippocampal and cortical regions-of-interest, spanning the imaged area projected through 30 1-micron optical sections, giving normalized densities across the samples.

#### MAP2 immunoreactivity analysis

Cross sectional tiled reconstructions of hippocampi stained for MAP2 were analyzed using Fiji/ImageJ. Grayscales were inverted, and thresholds set using an automated, user-independent Otsu/Huang thresholding algorithm. Defined rectangular regions of interest (ROIs) were placed over consistent areas of CA1, CA3 and dentate gyrus by an operator blind to treatment and age, and the percentage of pixels within the applied ROIs were measured, reflecting the staining density in the region.

### Statistics

Values throughout the manuscript are presented as mean ± sem unless otherwise indicated.

Statistical analysis was performed using Graphpad Prism software or SigmaStat. Sections were coded to reduce bias and Microbrightfield (MBF) unbiased stereology was used for the collection of cellular data. Temporal data was analyzed with one-way analysis of variance followed by post hoc comparisons. Analysis of the primary comparison of LM11A-31 treated mice to vehicle treated mice was done by t test. Additional t tests were run to verify changes in the aged mice relative to young mice or to examine effects in cholinergic subregions. Analyses of multiple regions were done using analysis of variance followed by post hoc comparisons. Specific tests are indicated for each experiment.

## Results

The most consistently reported age-related cellular changes involving BFCNs are loss of cell volume and neurite processes^10^ while reports of a decline in cell number are more variable^28,75^. In an aging model in which cell number did decrease, p75 knockout had no effect^29^. In the present study, cell counts in the combined MS/VDB region in a cohort of 4- and 18-month old mice were 5622 ± 1258 and 4479 ± 1077 (mean ± 95% confidence intervals). In a separate cohort of 4- and 25-month old mice, cell counts were 3806 ± 560 and 3038 ± 665. Thus, while there were decreases in cell counts at 18 and 25 months, these did not reach statistical significance. Considering these findings, the present study focused on measures of cell size and neurite processes as primary outcome measures.

**Figure 1:**
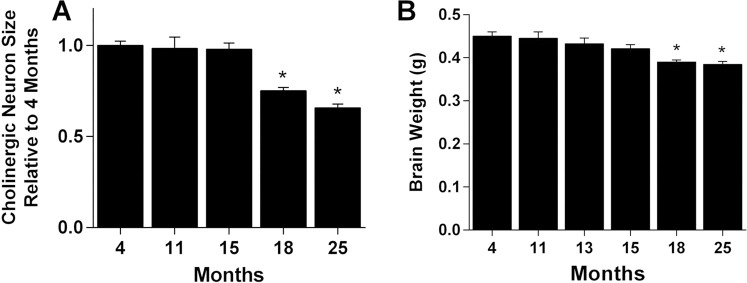
Age-related decreases in cholinergic neuron size and total brain weight. (**A**) Neuron areas in the medial septum (MS) and vertical diagonal band (VDB) at the indicated ages as a proportion of that in 4-month-old mice (*bars*, mean ± standard error of the mean (sem) of 6–12 mice). Decreases in size relative to young mice were significant at 18 and 25 months (p < 0.001, one-way analysis of variance with Dunnett’s multiple comparison test relative to 4-month-old mice at p < 0.05). (**B**) Brain weights at the indicated ages showed a gradual progressive decrease over time with significant decreases relative to young mice at 18 and 25 months. *(p < 0.001, one-way analysis of variance with Dunnett’s multiple comparison test relative to 4-month-old mice at p < 0.05). Values are expressed as mean + sem.

Age-related changes in the cross-sectional area of cholinergic cells within the medial septum (MS) and vertical diagonal band (VDB) nuclei were measured in mice using unbiased stereology at ages ranging from 4 to 25 months. The mean size of all cholinergic neurons at the 4-month reference age was 149.2 ± 5.5 µm^2^. Some regional variation was seen, with neurons of the VDB (162.5 ± 33.8 µm^2^) measuring 19.5% larger (p < 0.001, n = 34 mice) than neurons in the MS (136.0 ± 23.9 µm^2^). Significant shrinkage of cholinergic cells was first seen at 18 months (25.0 ± 1.8% loss) which progressed to 34.2 ± 2.0% by 25 months (Fig. 1A). No regional differences were seen in the timing or magnitude of the age-related decrease in cholinergic cell size. A small but progressive decrease in brain weight was seen with aging though this did not reach significance until 18 months, showing a 13.3 ± 1.0% decrease with a 14.6 ± 1.5% decrease by 25 months (Fig. 1B).

**Figure 2:**
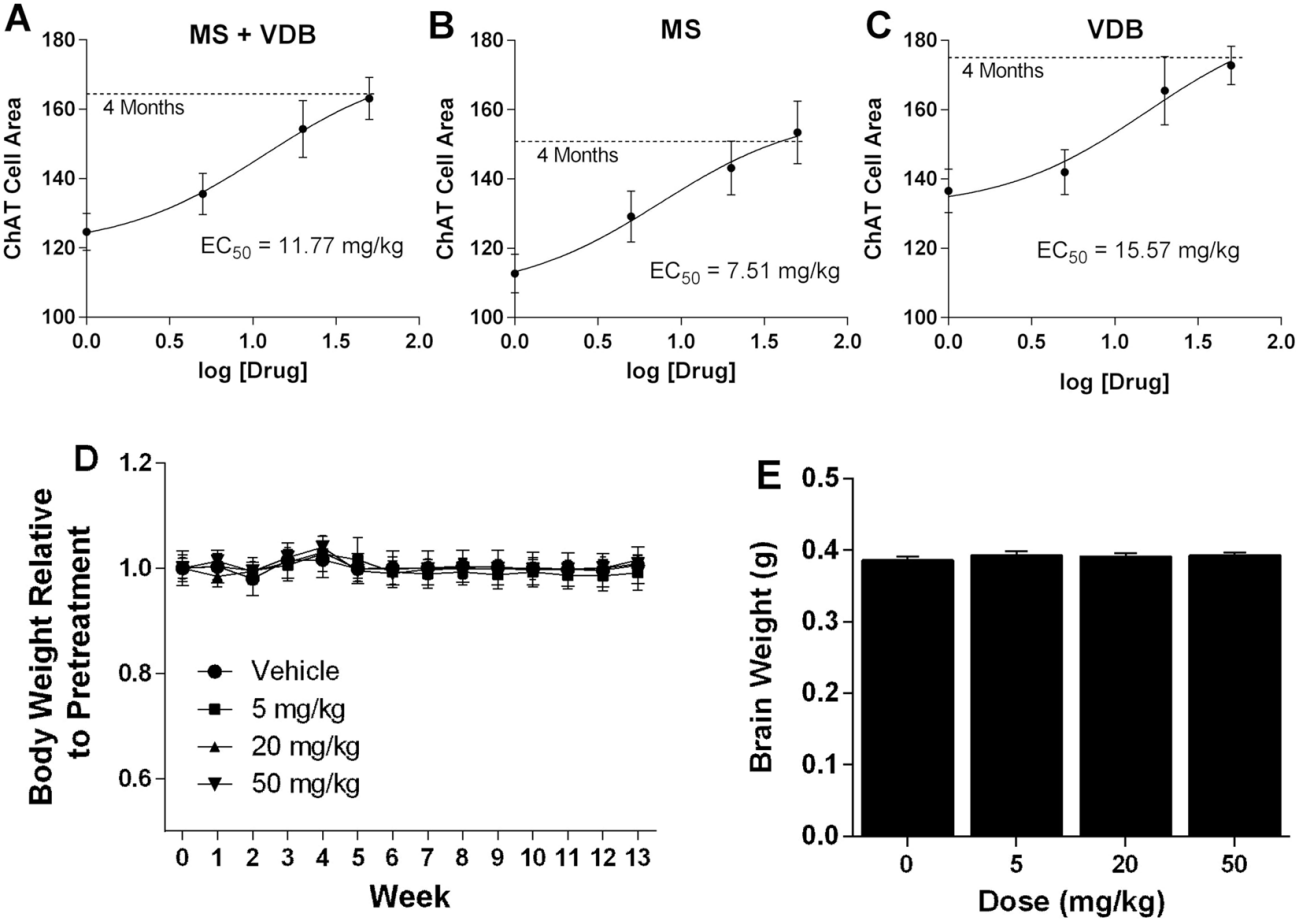
Drug dose-effect responses and systemic effects. (**A**–**C**) Area of ChAT immunoreactive cells in animals given LM11A-31 once daily for three months from age 15 to 18 months, at the indicated doses (n = 7–9 each). Values in a matched young cohort (age 4 months, n = 8) are indicated by the dashed line. Treatment inhibited the age-associated decrease in cholinergic neuron area in total (MS + VDB) and similarly in the MS and VDB individually. Composite data for all cholinergic cells (MS + VDB) yielded an EC_50_ of 11.77 mg/kg. (**D**) Mean body weights in each group as a proportion of pretreatment weights (31.4–33.0 g) did not change significantly over the course of the experiment. (**E**) Three-month treatment with LM11A-31 at 5–50 mg/kg had no effect on brain weights at 18 months. Values are expressed as mean + sem.

Effects of LM11A-31 on age-related changes in cholinergic cell morphology were first examined in a prevention paradigm, with daily treatment at 5, 20 or 50 mg/kg for three months beginning at 15 months and extending to 18 months, an age when significant changes would normally be seen. Cholinergic cell area in the MS + VDB (Fig. 2A), MS (Fig. 2B) and VDB (Fig. 2C) of LM11A-31 treated mice showed a dose-dependent increase in cholinergic cell area with maximal effects at 50 mg/kg, producing areas equivalent to those of 4-month old mice. The estimated EC_50_ from the composite data of these studies was 7.51 mg/kg for the MS, 15.57 mg/kg for the VDB and 11.77 mg/kg for the combined MS and VDB. Body weight was stable throughout the treatment (Fig. 2D) and there was no drug effect on brain weight (Fig. 2E).

**Figure 3:**
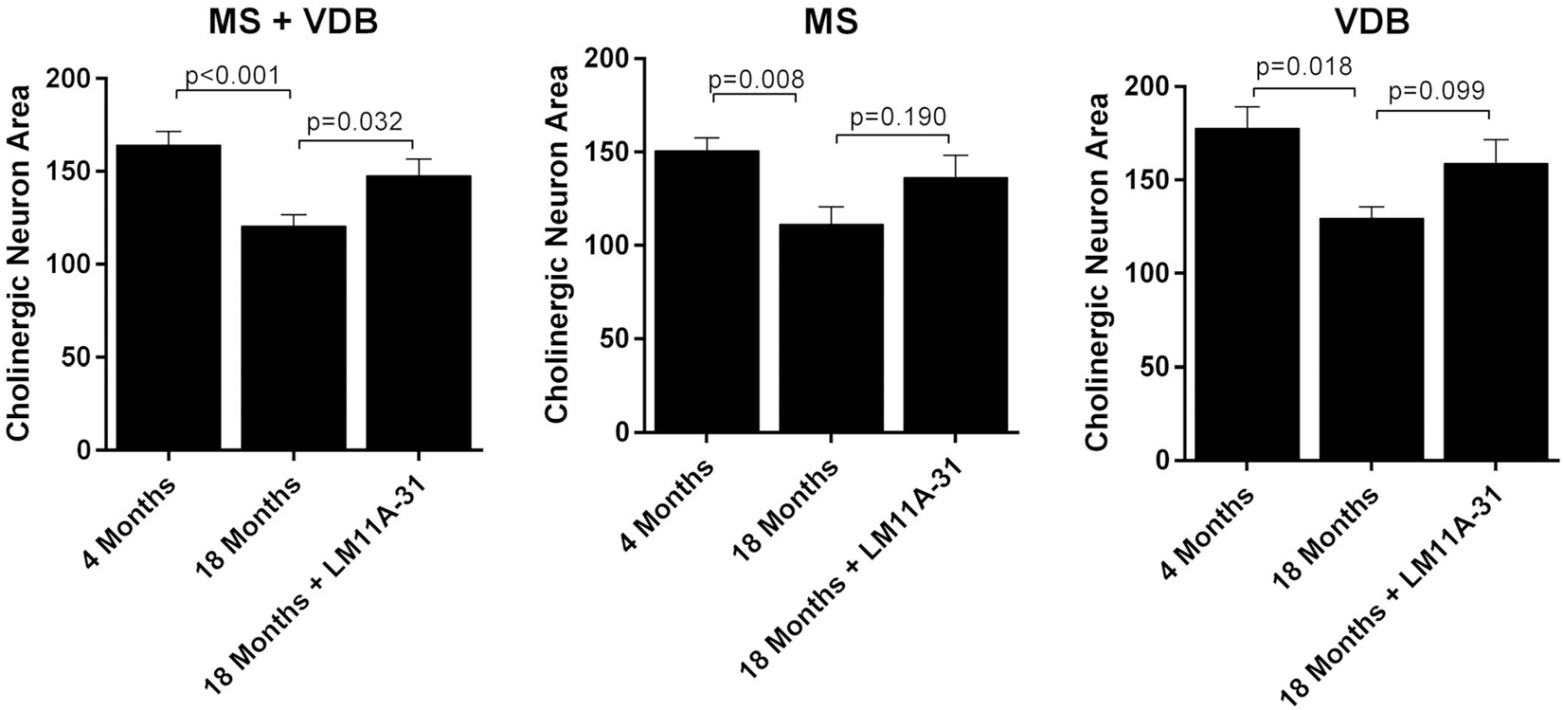
Diminished efficacy with shorter and later treatment. Five weeks of treatment once daily with 50 mg/kg LM11A-31 (n = 5) beginning at 17 months of age partially prevented and/or reversed the decrease in cholinergic neuron area in the MS and VDB seen in the vehicle treated mice (n = 4). Cholinergic neurons were identified by ChAT immunoreactivity and an unbiased estimate of area was obtained using Microbrightfield (MBF) software. A significant decrease in cell area measurements was seen in all groups (t-test, (n = 8) vs 18 months (n = 4), p values = 0.018–0.001). Inhibition and/or reversal of loss of cell size after five weeks of treatment was partial and reached significance only in the composite data for MS + VDB (t-test, treated vs vehicle, p = 0.032). Values are expressed as mean + sem.

A separate group of mice were treated with 50 mg/kg LM11A-31 for 5 weeks beginning at 17 months of age. Vehicle treated mice at 18 months showed a similar overall decrease in cholinergic MS/VDB cell area of 26.6% (MS + VDB) relative to young mice (4 Months) and no regional differences in the loss of cell area were seen within the MS (−26.1%) and VDB (−27.1%) (Fig. 3). LM11A-31 significantly reduced the overall loss of cholinergic cell area from −26.6 + 3.8% to −10.1 + 5.5% (MS + VDB) and though similar changes were seen in individual regions, these fell short of significance. A population analysis of the cholinergic cells (not shown) indicated that there was a small but significant shift toward larger cell area measurements in response to LM11A-31 in both the MS and VDB (X^2^, p’s < 0.001).

**Figure 4:**
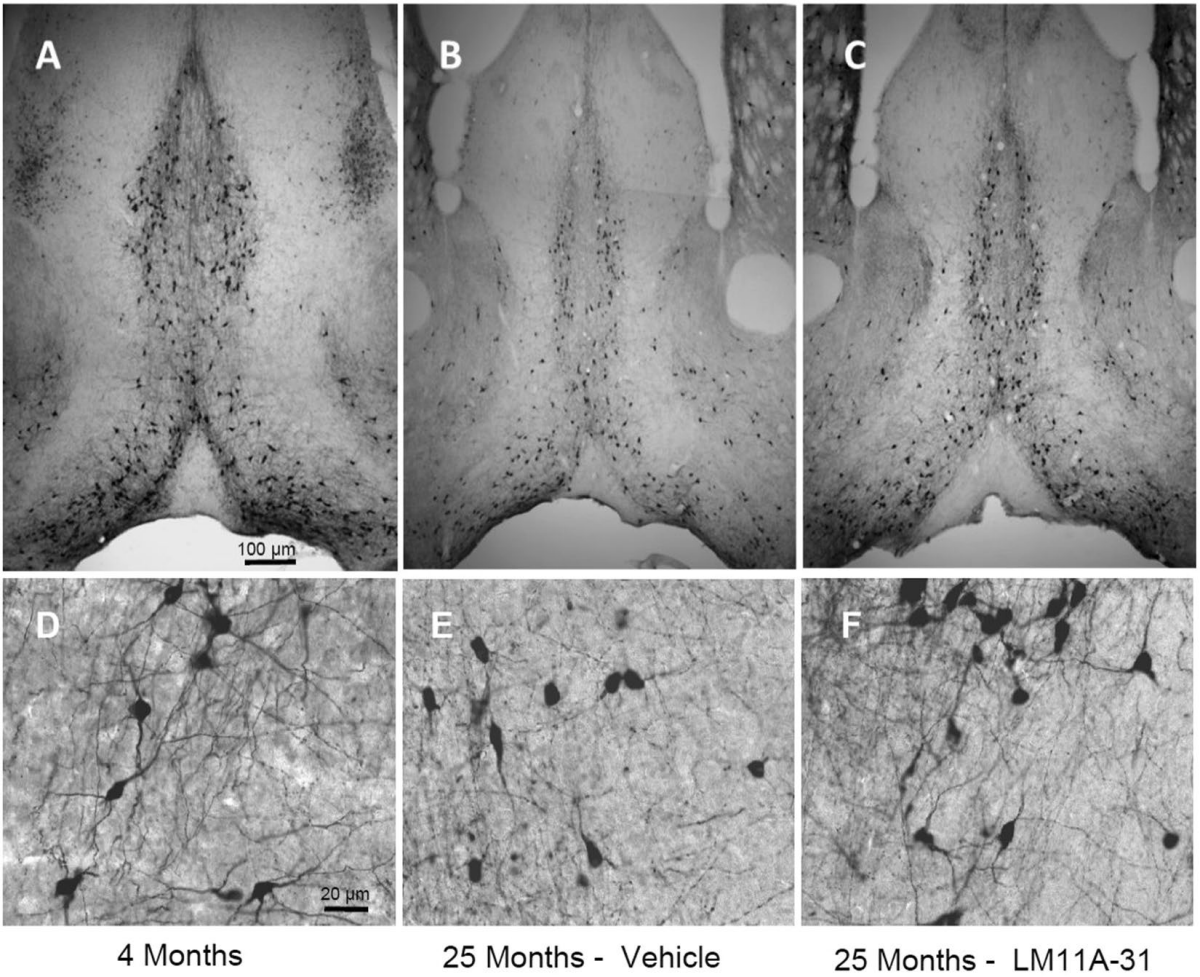
LM11A-31 efficacy at older ages. Representative choline acetyltransferase (ChAT) immunostaining of neurons in the medial septum (MS) and vertical limb of the diagonal band (VDB) of young, 4-month-old mice (**A**), and 25-month-old mice treated once daily with vehicle (**B**) or 50 mg/kg LM11A-31 (**C**) for four months from 21–25 months. Decreased cell size is apparent in 25-month-old animals, which is partially restored after treatment with LM11A-31. At higher magnification, neurons in the aged mice (**E**) have a shrunken appearance with a decrease in the size and extent of the neuritic network relative to young mice (**D**). Aged mice treated with LM11A-31 for 4 months (**F**) show partial restoration of cell size and neurites.

**Figure 5:**
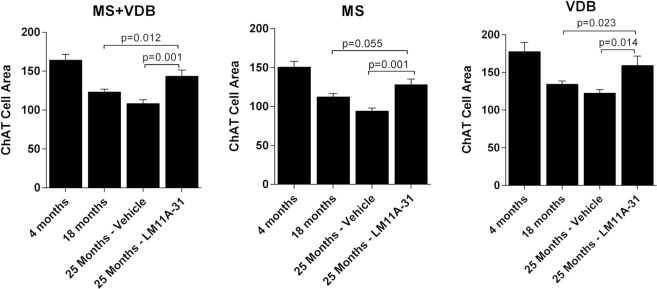
Restoration of established neuronal atrophy. Quantification of cholinergic neuron cell areas in the MS and VDB of 25-month-old mice (n = 12) showed significant losses of 37.5% in the MS and 31.0% in the VDB relative to that measured in 4-month-old mice (n = 8). With LM11A-31 administration for 4 months beginning at month 21, the size of cholinergic neurons was significantly greater in both regions reaching 85–90% of the size in young mice (t-test of treated vs vehicle, p’s = 0.014–0.001). Cholinergic neuron size in 25-month-old mice treated with LM11A-31 was also significantly greater than that in 18-month-old vehicle treated mice suggesting a reversal of atrophy relative to the age at which treatment was initiated (t-test of treated 25-month-old mice vs 18-month-old untreated mice). Values are expressed as mean + sem.

To examine the effects of LM11A-31 on established, advanced-stage cholinergic atrophy, mice were treated for four months with 50 mg/kg LM11A-31 beginning at 21 months of age. Within dendritic fields, overall ChAT staining appeared diminished in 25-month-old relative to young mice (Fig. 4A,B) and this loss of immunoreactivity appeared to be principally due to a decreased density of processes and shrinkage of neurons, rather than loss of neurons. LM11A-31 treatment partially restored cholinergic staining (Fig. 4C). In the aged mice, some neurons were shrunken in appearance, displayed truncated neurites (arrowhead), prominent varicosities (arrows) and an overall loss of neurite density relative to young mice (Fig. 4D,E). Following treatment with LM11A-31, neurons appeared less shrunken, the processes were less damaged and fewer varicosities were seen (Fig. 4F). At 25 months of age, the mean area of all cholinergic cells (MS + VDB) was decreased by 34.0 ± 3.0% (Fig. 5C) with roughly equal contributions from the MS (−37.5 ± 2.6%) (Fig. 5A) and VDB (−31.0 ± 2.6%) (Fig. 5B). The mean cell area was significantly restored to within 12.6 ± 4.8% of the size present in the four-month-old mice, with no significant regional differences. To further examine the degree of recovery, mean cholinergic cell area in mice receiving LM11A-31 from 21 to 25 months was compared to cell areas in untreated 18-month old mice, corresponding to the age of treatment initiation. Overall, the cholinergic cell size within the MS and VDB of 25-month old mice treated with LM11A-31 was significantly greater than the area in untreated 18-month-old mice (Fig. 5A). Similar patterns were observed in the MS (Fig. 5B), with borderline significance in the VDB (Fig. 5C).

**Figure 6:**
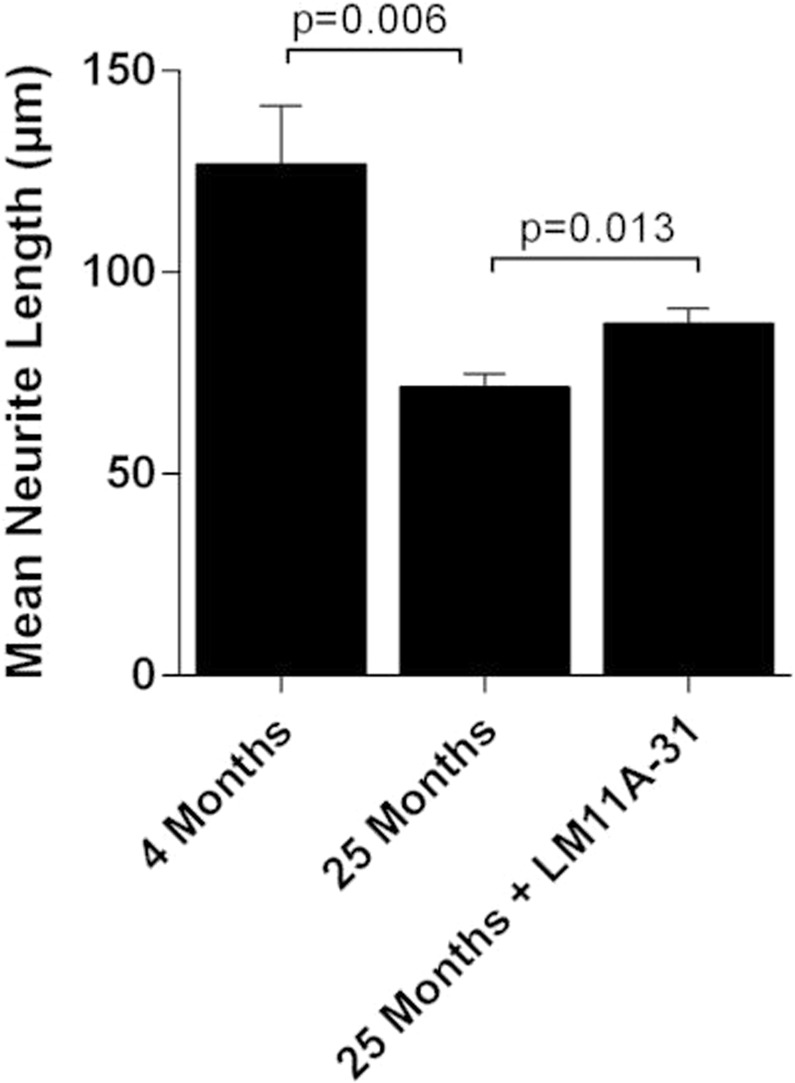
Treatment effects on neurite lengths. Quantification of neurite length within the basal forebrain (as in Fig. 4) showed a significant decrease in 25-month-old mice (n = 6) relative to 4-month-old mice (n = 6), from a mean of 126.8 ± 14.5 µm to 71.6 ± 3.2 µm (t test, 4 vs 25 months). In 25-month-old mice treated with 50 mg/kg LM11A-31 from 21 to 25 months, mean length (87.3 ± 3.8 µm) was significantly greater than the vehicle treated 25-month-old mice (p = 0.0131, t test, treated vs vehicle) and was 72% of the length seen in young mice. *Bars*, mean + sem.

In addition to cell shrinkage, aging was associated with loss of neuronal processes. To evaluate the extent of neurite loss and the effect of LM11A-31, the length of individual neurites in 25-month old mice treated with vehicle or LM11A-31 were compared to young 4-month-old mice and to each other (Fig. 6). In 25-month-old mice, the mean neurite length was reduced to 71.6 ± 3.2 µm relative to 126.8 ± 14.5 µm in the young mice, a 43.5% decrease. The mean length of neurites in the 25-month old mice treated with 50 mg/kg LM11A-31 was 87.3 ± 3.8 µm, reflecting a 22.0% improvement over the vehicle-treated, aged mice.

To determine whether treatment with LM11A-31 influenced MS/VDB cell number, estimates of cell numbers were compared between vehicle and LM11A-31 after 3 months of treatment (50 mg/kg) in 18-month-old mice and after 4 months of treatment in 25-month-old mice. At 18 months, cell counts were 4479 ± 1077 and 3832 ± 618; and at 25 months were 3038 ± 665 and 3735 ± 841. Therefore, it appeared that LM11A-31 had no detectable effect on MS/VDB cell number.

**Figure 7:**
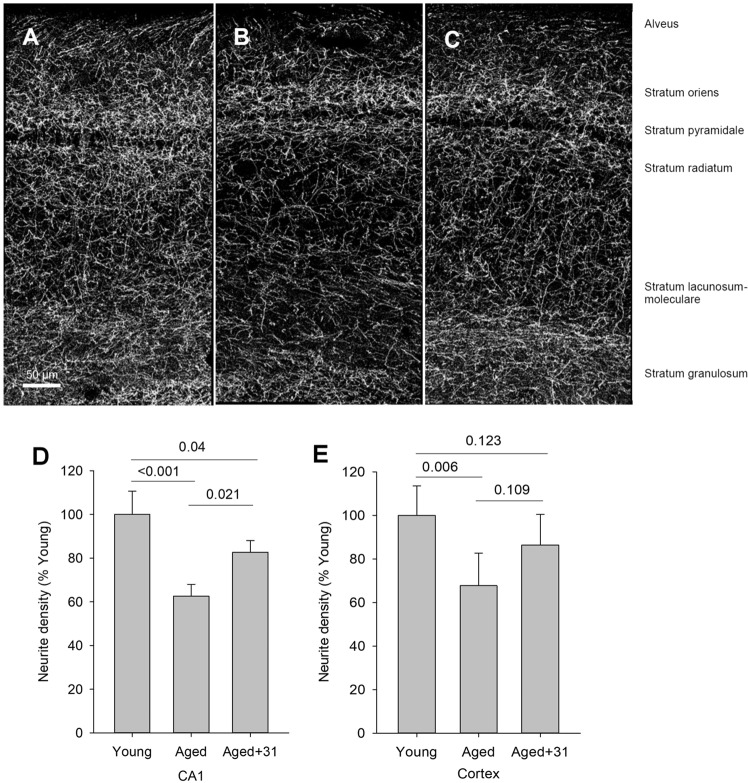
Hippocampal and cortical cholinergic fiber densities in young, aged and LM11A-31 treated/aged mice. (**A**) Representative image of ChAT-positive fibers in a coronal section of the hippocampus in the CA1 region of a 4-month-old mouse. (**B**) The greatest decreases in density in the aged mice (25 months) were apparent in the radiatum and lacunosum-moleculare. (**C**) Treatment with 50 mg/kg LM11A-31 from 21–25 months appeared to partially restore neurite density in the hippocampus. (**D**) Quantitation of neurite density in CA1 from a reconstruction of 30-image stacks with a region-of-interest spanning all layers showed a significant reduction in aged mice (25 months) relative to young mice (4 months) and partial recovery of density after treatment with LM11A-31. (**E**) Analysis of cholinergic fiber density encompassing layers 1–6 within the parietal cortex demonstrated a significant reduction with age. Treatment with LM11A-31 was associated with an increase in fiber density that did not reach significance. P values, as indicated above bars, are from a two-way ANOVA with factors of age/treatment and staining cohort using Holm-Sidak post-hoc analysis.

To evaluate the consequences of BFCN atrophy and neurite loss on distal targets which might correlate with age-related cognitive decline, cholinergic fiber density was examined in areas of the hippocampus and parietal cortex innervated by the MS and VDB in aged mice treated with vehicle versus 50 mg/kg LM11A-31. The hippocampus is considered to be a primary target of cholinergic innervation from the MS/VDB and the innervation of the CA1 region has been well characterized^76^. Young mice exhibit an extensive network of hippocampal cholinergic fibers with the highest densities in the oriens, radiatum and moleculare layers. In the present study, focused on CA1, the most notable loss of fiber density with age appeared in the radiatum and moleculare (Fig. 7A), layers that receive a high density of cholinergic input from the MS and VDB. Across all the layers of CA1 together, cholinergic fiber density showed an age-related decrease of 37.4 ± 8.6%, which was reduced to a 17.3 ± 10.3% with LM11A-31 treatment (Fig. 7D). In the parietal cortex, a significant age-associated decreased in cholinergic fiber density was also found (Fig. 7E) and treatment with LM11A-31 was associated with an increase in density which did not reach significance.

**Figure 8:**
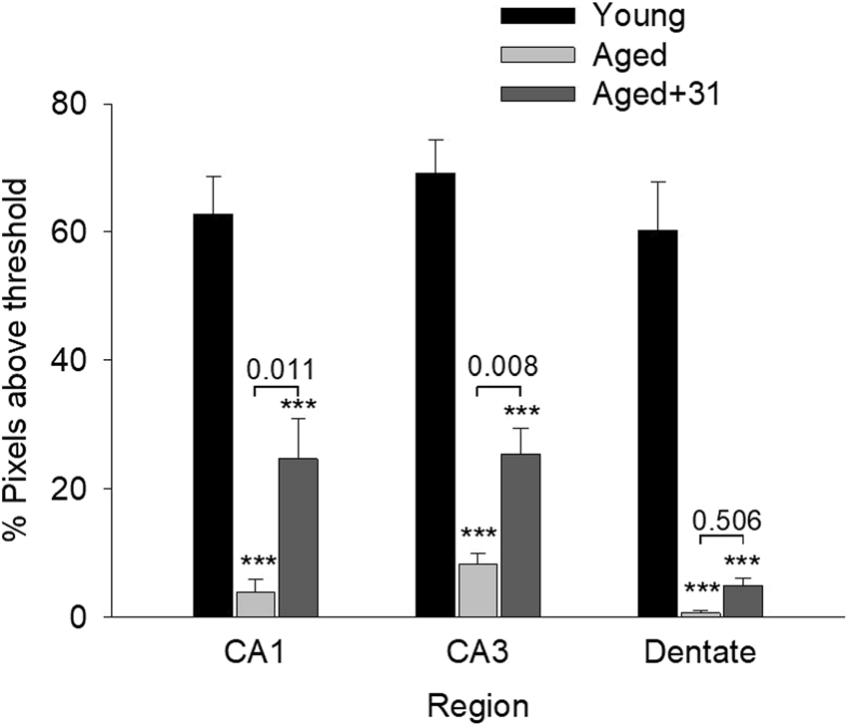
MAP-2 expression in young, aged and LM11A-31 treated/aged mice. The MAP-2 was imaged to eliminate background signal and provide a clean MAP-2 signal. The values shown therefore reflect relative MAP-2 intensities but not absolute expression. A significant loss of MAP-2 signal is observed in aged mice, which is partially recovered with LM11A-31 treatment, significantly in the CA1 and CA3 regions. ***P < 0.001 for comparison with young animals, other P values as indicated; N = 6 animals/group; ANOVA with post-hoc Student-Newman-Keuls testing.

Expression of the microtubule associated protein MAP2, a marker for neurites associated with hippocampal pyramidal and other cells, has been reported to be sensitive to axonal injury^77^ and to be markedly downregulated in aged rodents^78,79^. Similar marked decreases in MAP2 levels were found in hippocampal CA1, CA3 and dentate gyrus regions of 25-month-old mice relative to a 4-month-old cohort (Fig. 8). Treatment with LM11A-31 resulted in partial but significant recovery of MAP-2 levels in the CA1 and CA3 regions.

**Figure 9:**
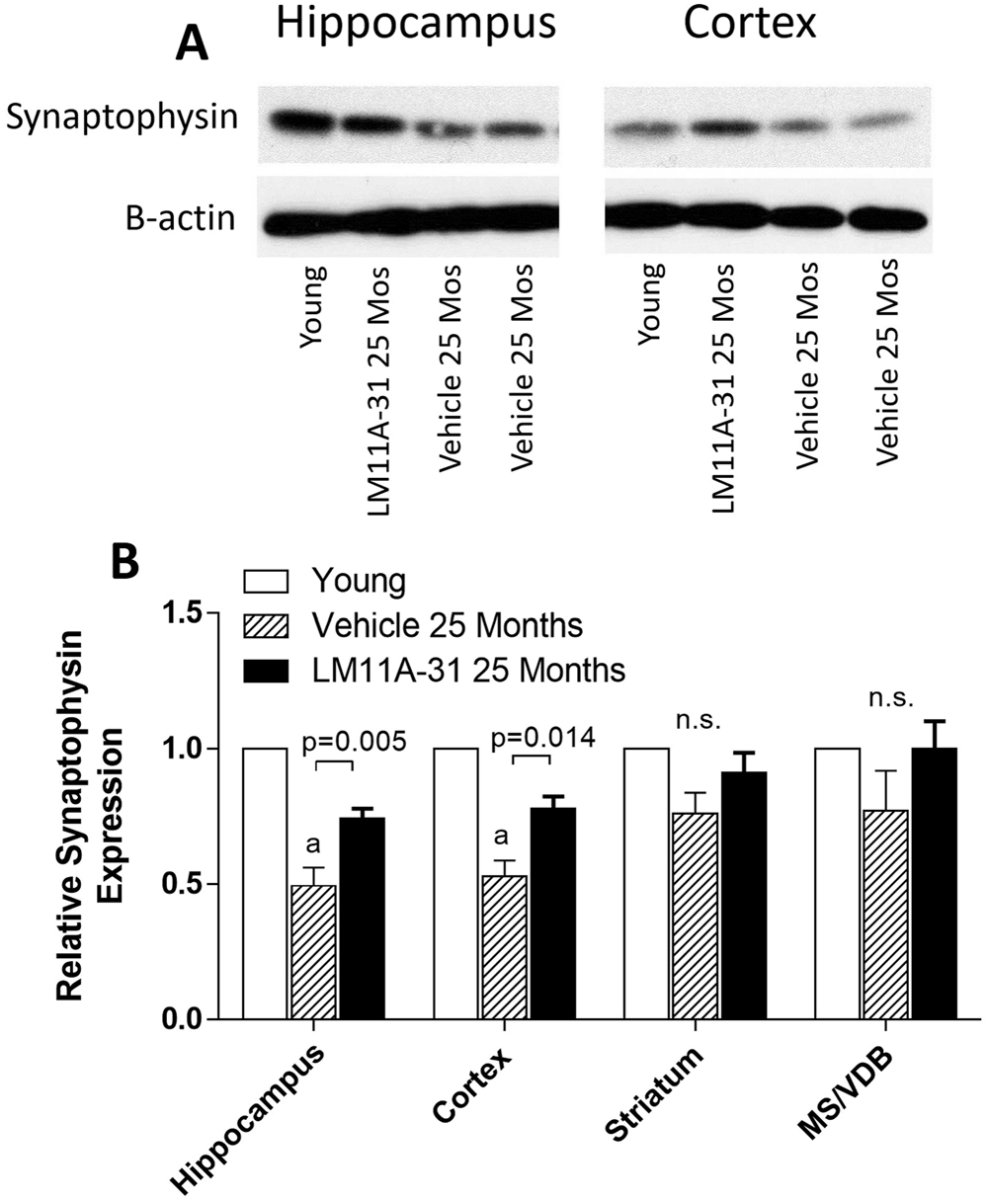
Western blot analysis of synaptophysin levels in the hippocampus of 4-month-old mice and 25-month-old mice treated with 50 mg/kg LM11A-31 or vehicle (saline) from 21 to 25 months. (**A**) In the hippocampus and cortex, synaptophysin was reduced in aged mice relative to young mice and partially restored by treatment with LM11A-31. The bands shown are representative of the differences seen across nine different blots and were acquired from the gel scans in Supplemental Fig. S1. Beta actin bands were generated from the same blot after stripping and re-probing. (**B**) Synaptophysin signal normalized to beta-actin showed significant age-related reduction in hippocampus, cortex and striatum with a significant recovery of signal associated with LM11A-31 treatment in the hippocampus and cortex (p’s = 0.005–0.014, t test, treated vs vehicle, n = 9). Values are mean + sem. *a*, P < 0.001 relative to young mice.

To determine if the age-related loss of cholinergic fiber density was associated with a loss of presynaptic termini, western blot analysis for the pre-synaptic marker synaptophysin was performed on hippocampus, cortex, striatum and basal forebrain tissue. Synaptophysin expression in the hippocampus was decreased in 25-month old mice to 49.4 ± 6.8% of that in 4-month-old (young) mice (Fig. 9). Treatment with LM11A-31 for four months beginning at 21 months, partially prevented the loss of synaptophysin to a level of 74.1 ± 3.8% of young mice. A similar effect was seen in the cortex (53.0 ± 5.8% of control; to 78.0 ± 4.5% with LM11A-31). A less robust loss of synaptophysin was seen in the striatum in the aged mice (76.1 ± 7.7% of young mice) which increased with LM11A-31 (91.3 ± 7.2% of young mice) but failed to reach significance due in part to the smaller initial deficit and greater variation. No significant changes in synaptophysin expression were seen in the ventral forebrain containing the MS and VDB although a similar pattern was noted.

## Discussion

The present study demonstrates an age-associated decrease in BFCN area and loss of neurite length with no significant changes detected in BFCN number. It is important to acknowledge that some but not all prior rodent studies have identified such age-associated markers of degeneration. This variation in findings across studies might involve differences in animal strains and housing, measurement techniques and time points evaluated^10^.

Several lines of evidence have suggested that p75^NTR^ related mechanisms contribute to age-associated BFCN degeneration. Al-Shawi *et al*.^38^ found that application of proNGF to brain slices derived from young (3–6 mo) mouse brain had no effect on BFCNs, but when applied to slices from aged (22–30 mo) mice caused substantial neuronal death. These investigators also found increased levels of proNGF in hippocampal but not cortical tissue harvested from both aged mice (22–20 mo) and aged rats (22–30 mo). Within mouse basal forebrain, no age-related increase in p75^NTR^ levels was detected. In contrast, in another study^35^, an increase in basal forebrain p75^NTR^ levels was detected in aged rat brain. In this same study, a single injection of proNGF into the hippocampus of aged rats led to BFCN cell body atrophy along with a decrease in the density of neurites in the region. In studies of human hippocampus and adjacent cortex, p75^NTR^ immunostaining was found to increase into the fourth decade^80^. Other studies have found that levels of basal forebrain TrkA are reduced with aging with a concomitant increase in the ratio of p75^NTR^ to TrkA expression within BFCNs, which may be a particularly potent driver of neural degeneration^81–84^. Cholinergic degeneration, in particular, is likely to be highly vulnerable to the combination of age-related increased p75^NTR^, decreased TrkA and increased levels of proNGF^34,81^. In rats, age-related deficits in spatial learning and recognition memory have been associated with increased levels of proNGF and p75^NTR^ in the frontal cortex and hippocampus^39^. Decreases in mature NGF gave rise to approximately a 4-fold increase in the proNGF:NGF ratio in aged rats. In addition, reductions in dendrite complexity and spine density have been seen in hippocampal neurons overexpressing p75^NTR^^85^. The present studies, showing that an agent which modulates p75^NTR^ activity slows, and may reverse, effects of aging on basal forebrain neurons, further support the view that p75^NTR^ and its ligands may play an important role in aging-related pathology and dysfunction of that brain region.

LM11A-31 effects on age-related degeneration could occur through several non-mutually exclusive mechanisms. These include modulation of p75^NTR^ signaling to upregulate trophic mechanisms such as recruitment of the IRAK adaptor and promotion of AKT signaling^64^ and to downregulate degenerative mechanisms including JNK and excessive RhoA activation^64,67,68^. Characterization of LM11A-31 has shown it to prevent death of cultured neurons through p75^NTR^-dependent mechanisms in the absence of pro- and mature NTs. In addition, LM11A-31 has been shown to inhibit the ability of proNGF to induce cell death when added to oligodendrocyte cultures^64^, to inhibit proNGF binding to p75^NTR^ in an *in vivo* model of spinal cord injury in which proNGF levels are raised^69^, and to inhibit proNGF/p75^NTR^-induced bladder dysfunction also following spinal cord injury^86^. Thus, through these two mechanisms, LM11A-31 might reset the balance of neurotrophin signaling in aging by offsetting the effects of an increased p75^NTR^/TrkA ratio in association with increases in proNGF levels. This could be expected to have potent protective effects through suppressing early processes associated with age-related neurodegeneration. Moreover, even if p75^NTR^-mediated mechanisms are not a primary driver of degeneration, LM11A-31 may be effective through activation of signaling that favors survival and preservation of structural integrity in cells expressing the receptor.

The effects of LM11A-31 on preventing or reversing age-associated degeneration of BFCNs and their projections likely involves a direct effect through their p75^NTR^ receptors. However, LM11A-31 effects on degenerative changes in cells intrinsic to the hippocampus such as pyramidal neurons have been found in a model of Huntington’s disease^72^. Thus, reversing age-related degeneration could be either a direct effect through p75^NTR^ expressed by pyramidal neurons^87^ or through an indirect result of enhanced cholinergic fiber input. The finding that LM11A-31 treatment stimulates a recovery of the synaptophysin presynaptic marker is consistent with the latter mechanism although the two mechanisms are not mutually exclusive.

While aging is generally considered to be the most powerful risk factor for the onset of AD, the extent to which age-related mechanisms promote or are additive to AD, versus the less likely alternative that AD mechanisms simply require long durations, remains to be further defined. The finding that BFCNs in older versus younger rats are significantly more vulnerable to intraventricular injections of amyloid beta (Aβ25–35), supports a model for age-related processes contributing to AD mechanisms^88^. Further supporting this notion is the finding that injections of fibrillar Aβ in cortical tissue led to markedly greater loss of cortical neurons in older versus younger non-human primates^89^. The potential role of aging processes in AD cognitive impairment raises the possibility that candidate AD therapies are more likely to be effective if they directly address age-related degeneration along with AD-linked mechanisms, such as those involving amyloid and tau. The present study, combined with our prior studies in AD models, indicates that p75^NTR^ small molecule ligands may constitute a class of therapeutics capable of engaging both categories of degenerative mechanisms to mitigate the effects of aging.

## Supplementary information

Supplementary Information

## Data Availability

The data generated during and/or analyzed during the current study are available from the corresponding author on reasonable request.
