# Supplementary material for: Modulation of the p75 neurotrophin receptor suppresses age-related basal forebrain cholinergic neuron degeneration
Source: Sci Rep. 2019 Mar 27;9:5273. doi: 10.1038/s41598-019-41654-8 (PMC6437186; doi:10.1038/s41598-019-41654-8)

Modulation of the p75 neurotrophin receptor suppresses age-related basal forebrain cholinergic neuron degeneration

Youmei Xie, Rick B. Meeker, Stephen M. Massa, Frank M. Longo

Fig S1.
Full length western blot scans utilized in Fig 9.
First: Synaptophysin
Second: actin

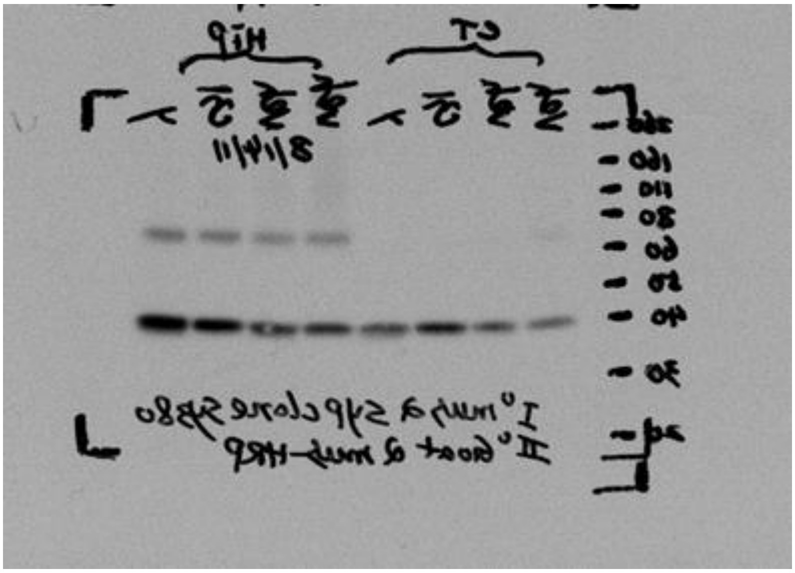

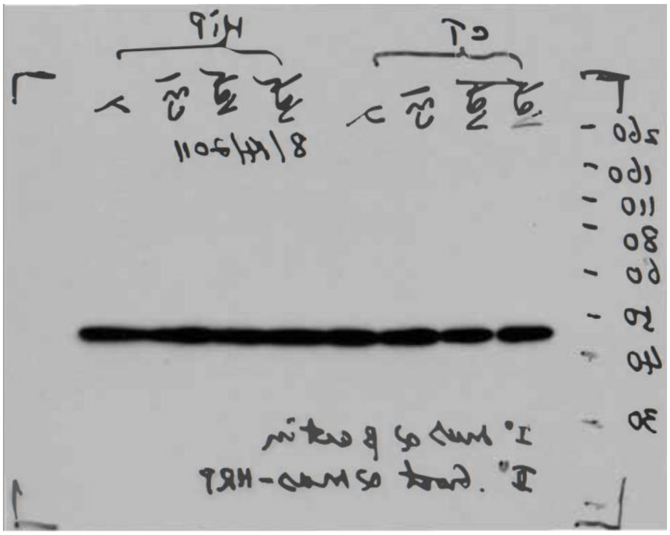

Supplement: Supplementary file 1 — Supplementary Information [file 41598_2019_41654_MOESM1_ESM.pdf]
